# UNESCO and the history of anti-racism in postwar science

**DOI:** 10.1590/S0104-59702025000100012

**Published:** 2025-04-07

**Authors:** Marco Antonio Ramos

**Affiliations:** i Assistant Professor, History of Science and Medicine/Yale University. New Haven – CT – USA, orcid.org/0000-0002-1649-7547, marco.ramos@yale.edu

**Figure:**
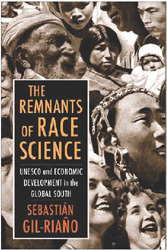
GIL-RIAÑO, Sebastián. *The remnants of race science: UNESCO and economic development in the Global South*. New York: Columbia University Press, 2023. 392p.

Campus activism and the recent surge of academic interest in the history of science and race, especially through the study of slavery and eugenics, has pushed universities to reckon with their legacies of racism (Willoughby, 2022). A variety of “anti-racist” programs have emerged that claim to challenge racial discrimination, from mandatory implicit association testing to university-sponsored historical research on slavery and offices of diversity, equity, and inclusion.

Sebastián Gil-Riaño’s *The remnants of race science: UNESCO and economic development in the Global South* is essential reading for historicizing anti-racism within and beyond the university. Gil-Riaño’s book focuses on the history of the United Nations Scientific, Educational and Cultural Organization (UNESCO) and its attempts to use science to combat racial discrimination around the globe during the postwar period. *Remnants of race* upends the all-too-common narrative about the decline of scientific racism in the twentieth century. In this narrative, the overtly racist eugenic science that identified races with distinct, fixed physical traits was renounced by scientists who committed to fighting racial discrimination in the wake of Nazi fascism. As Gil-Riaño (2023) points out, this “narrative of redemption,” which was reflected and reinforced in UNESCO’s controversial 1950 and 1951 Statements on Race, functioned to absolve contemporary science of the misguided and “unscientific” racism of the past.

*Remnants of race* challenges this narrative and offers a new understanding of the history of racism, anti-racism, and science in at least two ways. First, Gil-Riaño demonstrates that human science did not reject the exploration of racial difference in the postwar period wholesale. Instead, racialized reasoning was incorporated into new discourses of global economic development. *Remnants of race* traces how “hard eugenic” racial science of physical anthropologists from the early twentieth century made way for more social and cultural approaches to race and racial discrimination advanced by social anthropologists, social psychologists, and sociologists. Focusing on the Swiss-born and French-trained anthropologist Alfred Métraux, who served as the director of UNESCO’s race campaign in the 1950s, the book explores how social anthropologists emerged as the authoritative international experts on race in the postwar period. However, while these social scientists rejected the physicalist assumptions of earlier twentieth-century eugenic science on the grounds that it was “unscientific,” their newer social approaches also reproduced and reinforced racist colonial logics through discourses of development that infantilized non-Europeans because of their supposed lack of societal progress. As Gil-Riaño (2023) demonstrates in cases throughout the Global South, UNESCO scientists claimed to fight racism by arguing that “isolated” Indigenous societies in the peripheral “dark zones” of the world needed to be assimilated, acculturated, and developed to Western standards of civilization. These developmentalist arguments justified the forced removal and assimilation of Indigenous children and the postcolonial incorporation of “undeveloped” societies into global capitalism. Exposing the racialized social science that undergirded postwar development, *Remnants of race* facilitates a long-overdue conversation between the critical literature on international development and histories of race in the global twentieth century.

Second, *Remnants of race* suggests that postwar science on race and racial discrimination was not a one-way ticket from scholars in the Global North to projects in the Global South. Building on recent work that has privileged perspectives and theory-making from the South, Gil-Riaño (2023) explores how scientists, especially in Latin America, developed more flexible ideas of racialized human difference that attended to culture, social organization, and economic conditions long before their adoption by internationalist groups like UNESCO (Connell, 2020). For example, the head of UNESCO’s Social Sciences Department, Brazilian anthropologist and psychiatrist Arthur Ramos, advanced a more flexible and sociocultural approach to race and culture that was grounded in an older, distinctly Bahian approach to anthropology. For Gil-Riaño (2023), the postwar sociocultural understanding of human difference was consequently not simply a break from European histories of racist eugenic science, but rather was continuous with a longer history of more flexible and dynamic racial thought in Latin American science (Stepan, 1991).

The book is divided into three parts that follow a rough chronology from 1920 to 1960. Part I traces the impact of Latin American anthropology, especially from Brazil, on the development of the UNESCO Statements on Race at midcentury. Part II connects sociocultural approaches to race with developmentalist discourse, through the examination of two ambitious UNESCO projects from the 1950s: the International Institute of the Hylean Amazon and the Andean Indian Program in Perú. The Final Part examines UNESCO’s efforts to construct “race relations” as a field of inquiry intended to identify and spread portable sociological models of “racial harmony” around the world. As Gil-Riaño (2023) argues, many researchers in the Global South ultimately rejected “race relations” as a field because it attempted to stabilize, instead of challenge, an unjust global order. The engaging conclusion demonstrates how Métraux’s work on salvage ethnography, which presumed the need to “salvage” Indigenous cultures because of their inevitable collapse, began to articulate with later biological efforts to rescue the genetics of Indigenous communities, in controversial programs like the Human Genome Diversity Project.

*Remnants of race* is an invaluable contribution to the growing body of work exploring the new scientific racisms that have emerged in the aftermath of eugenics – often in the name of fighting racial discrimination itself (Alberto, Elena, 2016). Bringing attention to Southern thinkers who have traditionally been marginalized in the literature, the book artfully weaves together the history of anthropology, history of science, and studies of international development, modernity, and *indigenismo*. It is filled with nuance and gifted storytelling. *Remnants of race* will be critical not only for students and scholars in these fields, but also for organizers and activists grappling with the possibilities and limits of anti-racism in science today.
